# COST–RISK–BENEFIT ANALYSIS IN DIAGNOSTIC RADIOLOGY: A THEORETICAL AND ECONOMIC BASIS FOR RADIATION PROTECTION OF THE PATIENT

**DOI:** 10.1093/rpd/ncv506

**Published:** 2016-06-07

**Authors:** B. Michael Moores

**Affiliations:** Integrated Radiological Services Ltd, Unit 188, Century Building, Brunswick Business Park, Liverpool, UK

## Abstract

In 1973, International Commission on Radiological Protection Publication 22 recommended that the acceptability of radiation exposure levels for a given activity should be determined by a process of cost–benefit analysis. It was felt that this approach could be used to underpin both the principle of ALARA as well for justification purposes. The net benefit, *B*, of an operation involving irradiation was regarded as equal to the difference between its gross benefit, *V*, and the sum of three components; the basic production cost associated with the operation, *P*; the cost of achieving the selected level of protection, *X*; and the cost *Y* of the detriment involved in the operation: B=V−(P+X+Y). This article presents a theoretical cost–risk–benefit analysis that is applicable to the diagnostic accuracy (Levels 1 and 2) of the hierarchical efficacy model presented by National Council on Radiation Protection and Measurements in 1992. This enables the costs of an examination to be related to the sensitivity and specificity of an X-ray examination within a defined clinical problem setting and introduces both false-positive/false-negative diagnostic outcomes into the patient radiation protection framework.

## INTRODUCTION

The question has been asked; ‘What has radiation protection of patients achieved since its inception and how can its effects be quantified?’^(1)^ A corollary to this question is whether the effects of radiation protection can in fact be quantified and if so how?

Such questions are not easily addressed given the multifaceted nature of the use of ionising radiation in healthcare particularly for diagnostic purposes. Because of this complexity, medical radiation protection in diagnostic radiology has focussed most attention on the doses delivered to patients and established a radiation risk-based framework for radiation protection. Unfortunately, purely radiation risk-based strategies have not prevented significant growth in both population and individual patient doses arising from radiological practices over the past four decades.

To some, this may be considered an acceptable healthcare strategy when high-quality diagnostic examinations are being made more widely available to the population. However, overdependence on technology-driven healthcare can be counterproductive, especially if it leads to non-selective overuse. Consequently, the appropriateness of diagnostic X-ray examinations and the underlying principle of justification are currently receiving much attention. The role of clinical judgement prior to an examination, representing a clinical selection process, is a primary consideration when escalating healthcare budgets can result from a strategy of easy or open access.

Radiation risk-related radiation protection strategies apply unequivocally to protection of workers and the general public; however, they are less suited to protection of patients who are exposed directly as part of diagnosis or treatment. This is especially true when the actual doses and associated risks that arise from most diagnostic X-ray examinations are very low (e.g. in the dose range <10 mSv). On the other hand, the clinical risks associated with not performing a satisfactory examination may be significant either from the perspective of quality of life or even death of a patient. This latter fact and an associated fear of litigation have helped to drive the continuous growth in the use of diagnostic radiology worldwide.

During the early development of the system of radiation protection, aimed mainly at workers, purely radiation risk-related (defensive) strategies took precedence with dose limitation a primary driving force. This took place when early pioneers of X-ray applications were still dying from injuries received. However, throughout the 1960s, as strategies for medical applications came to the fore, a broader-based approach evolved. Consequently, International Commission on Radiological Protection (ICRP) Publication 9 published in 1965 stated^(2)^:

As any exposure may involve some degree of risk, the Commission recommends that any unnecessary exposure be avoided, and that all doses be kept as low as is readily achievable, economic and social considerations being taken into account.

Development of the newly stated ALARA principle with its economic and social considerations culminated in the 1973 ICRP Publication 22, which recommended that the acceptability of radiation exposure levels for a given activity should be determined by a process of cost–benefit analysis^(3)^. It was felt that this approach could be used to underpin both the principle of ALARA as well as for justification purposes.

ICRP Publication 22 stated that benefits were likely to be both tangible, identifiable and subject to quantification in terms of monetary or other units and intangible, which contribute to the satisfaction of human desires but not subject to formal quantification. Costs were defined as the sum total of all negative aspects of a given operation including manufacturing, operating and maintaining as well as all other expenses, losses, liabilities and induced adverse effects, whether tangible or intangible. The latter included any effects contributing to human unhappiness.

This approach was initially applied to processes where a practice that employed ionising radiation could be separated (theoretically at least) from the irradiation of the population. However, for medical practices, patients are exposed directly in order to achieve any benefit. Equally, benefits arising from medical applications are somewhat open ended, for diagnostic applications at least. Any overall benefits from a diagnostic outcome might accrue after a number of subsequent clinical stages in a patient pathway. These may enhance or equally degrade the diagnostic outcome depending upon the relevance and/or quality of subsequent clinical actions. Consequently, the efficacy of a diagnostic process forms part of a multilayered clinical process.

In 1991, National Council on Radiation Protection and Measurements (NCRP) published a commentary on efficacy in diagnostic radiology and nuclear medicine aimed at underpinning the concept of justification of medical radiation exposure^(4)^. Efficacy was defined as the probability of benefit to individuals in a defined group of patients from a medical technology applied for a given medical problem under ideal conditions of use. Hence, fundamental to this definition is the concept that variations in any defined patient group undergoing an investigation of a given medical problem might affect the efficacy. Equally the application of a technology under less than ideal conditions might also affect efficacy. In fact, efficacy was indicated to be an essential component of radiation protection in medicine in that it provides the basis for evaluating whether a procedure or practice is justified. If efficacy can vary with such fundamental conditions then so too must any underlying justification. Consequently in routine and variable practice, justification cannot be taken as a uniquely defined ethical entity^(1)^ but will be linked to a spectrum of clinical, safety and economic considerations and may even be a statistical variable.

The efficacy model presented by NCRP has six hierarchical levels of efficacy^(4)^:

**Level 1**. Technical efficacy—resolution, modulation transfer function, greyscale range, noise, sharpness, patient dose, etc.
**Level 2.** Diagnostic accuracy efficacy—yield of abnormal or normal diagnoses, sensitivity and specificity in a defined clinical problem.
**Level 3.** Diagnostic thinking efficacy—the number (percentage) of cases in a series in which image judged helpful to making the diagnosis, entropy change in differential diagnosis probability distribution.
**Level 4.** Therapeutic efficacy—the number (percentage) of times image judged helpful in planning management of the patient including avoidance of medical procedure.
**Level 5.** Patient outcome efficacy—percentage of patients improved with test compared with those improved without including morbidity avoided.
**Level 6.** Societal efficacy—cost-effectiveness analysis from societal viewpoint.

Obviously, the scope of any cost–benefit analysis must be dictated by the overall efficacy framework that is applied. Within the NCRP model, the first two efficacy levels apply directly to the diagnostic imaging process (production and interpretation of images), with higher levels taking cognisance of other clinical patient pathway components. Consequently, this present work will confine the theoretical cost–risk–benefit analysis within the purely diagnostic process up to Level 2, the Diagnostic Accuracy Efficacy of the NCRP hierarchical model. Level 1, technical efficacy concerned with the physical performance of the imaging system underpins this Level 2 efficacy and should, hopefully, have a bearing on it.

## THEORETICAL BACKGROUND

According to the ICRP cost–benefit model, the net benefit *B*, of an operation involving irradiation, was regarded as being equal to the difference between its gross benefit, *V*, and the sum of three components; the basic production cost associated with the operation, *P*, the cost of achieving the selected level of protection, *X*; and the cost *Y* of the detriment involved in the operation^(3)^. Thus,

(1) B=V−(P+X+Y)

This approach has been applied previously to the optimum quality control of gaseous tritium light sources intended for use in liquid crystal display digital watches^(5)^. In this case, both *V* and *P* can be considered to be independent of exposure. However, for medical practices where patients are exposed directly to achieve any benefit, this is not necessarily the case. Also, in order to maintain a degree of simplicity, it is reasonable to assume a single X-ray unit undertaking a specific type of examination on a given group of patients *N*, over a defined period of time, such as its amortised lifetime. However, the model may easily be extended to include multiple units and modalities undertaking a range of different types of examinations on a variety of different definable patient groups over any selected time period.

For a given level of exposure *E*, the production costs *P*(*E*) are given by

(2) P(E)=M(E)+I(E)+Serv(E)+Op(E)

where *M*(*E*) is the manufacturing and sales (i.e. purchase) costs, *I*(*E*) is the installation costs, Serv(*E*) is the service and maintenance costs and Op(*E*) is the direct operating costs including staff and overhead throughout any chosen period of time. For generality, it has been assumed that these are exposure dependent since equipment employing higher-dose techniques tend to be more expensive to purchase and operate; however, this will not affect the present analysis.

The cost of achieving a given level of protection, *X*(*E*), is dictated by the overall framework for radiation protection that is employed locally including the costs incurred in meeting any legislative requirements; manpower, training and optimisation costs throughout the chosen period. Again, these costs are assumed to be exposure dependent. Specific building costs to meet necessary safety requirements are assumed to be included in *P*(*E*).

The gross benefit *V* arising from X-ray examinations on a particular defined group of patients up to Level 2 efficacy is dictated by the true-positive *T*/*P*(*E*) and true-negative *T*/*N*(*E*) detection rates so that $V=(N_{P}T/P(E))+(N_{N}T/N(E))$. Here, *N*_P_ is the number of diseased patients, and *N*_N_ is the number of normal healthy patients in a total group *N* = *N*_P_ + *N*_N_, and the prevalence of disease will be given by $\text{Pr}=N_{P}/\left(N_{P}+N_{N}\right)$. Within the Level 2 framework of efficacy, the actual monetary value of gross benefits (the cost benefit) is dictated by the monetary costs of running a service, since this determines the cost of achieving true-positive and true-negative outcomes. Also, it is assumed that the cost benefits arising from both positive and negative diagnoses are the same at the Level 2 efficacy stage.

The cost detriment, *Y*(*E*) = *R*_X_(*E*) + *R*_D_(*E*), where *R*_X_(*E*) is the radiation cost detriment associated with the use of ionising radiation on a given group of patients, and *R*_D_(*E*) is the diagnostic cost detriment associated with a false diagnosis; *F*/*N*(*E*)—false negative and *F*/*P*(*E*)—false positive^(6)^. Consequently, $R_{D}(E)=(N_{P}F/N(E))+(N_{N}F/P(E))$ where *N* patients are examined throughout the chosen time period, and this would represent the overall diagnostic cost detriment. *R*_X_(*E*) = *NR*_X_(*E*) is the number of cancers induced with *R*_X_(*E*), the probability of cancer induction for a given patient dose*.* The cost detriment arising from rejected or repeat examinations would of course be included in *R*_X_(*E*).

As with the gross benefit within the Level 2 efficacy framework, the diagnostic cost detriment also arises from the overall costs of running a service. Any future effects and the associated costs of the incorrect diagnoses fall outside the initial/primary diagnostic process (Levels 1 and 2) efficacy framework. Thus, throughout the chosen time period, Equation 1 may be written:

(3) $$\begin{array}{ll} B(E) & =\left[N_{P}T/P(E)+N_{N}T/N(E)\right]-[P(E)+X(E)]\\ & \;-\left[NR_{X}(E)+N_{P}F/N(E)+N_{N}F/P(E)\right] \end{array}$$

The overall costs of running an X-ray unit can be equated for the period under consideration as *K*(*E*) = *P*(*E*) + *X*(*E*). However, the model could include non-uniform costs during the chosen period of time, for example as equipment ages its service and maintenance costs might rise.

The magnitude of any net benefits that might arise to society from true-positive and true-negative outcomes at the diagnostic efficacy Level 2 are unknown. Studies of outcomes at higher levels of efficacy would be required to deduce any net benefits that might be directly attributable to the diagnostic efficacy Level 2. Therefore, it is not unreasonable to assume that an X-ray unit operates at break-even at the purely diagnostic level and the net benefit *B*(*E*) = 0. Thus, all cost benefits arise out of actually providing a service. Rearranging Equation 3 gives

(4) $$K(E)=\left[N_{P}T/P(E)+N_{N}T/N(E)\right]-\left\{\left[N_{P}F/N(E)+N_{N}F/P(E)\right]\;+NR_{X}(E)\right\}$$

This equation represents the cost–risk–benefit framework for a representative single X-ray unit undertaking examinations on individuals in a ‘defined group of patients for a medical technology applied for a given medical problem under ideal conditions of use’. It expresses the net beneficial patient diagnostic outcomes (net cost benefit) when corrected for detrimental (false) outcomes in terms of observed mean values of diagnostic quantities that are statistical variables. Furthermore, if it is assumed that the idealised unit detects all abnormalities and verifies all negative findings perfectly, then (T/P(E)=T/N(E)=1) and *NR*_X_(*E*) ≪ *N*_P_, *N*_N_ (see later), then Equation 4 gives

(5) $$K(E)=\left[N_{P}T/P(E)+N_{N}T/N(E)\right]\;-NR_{X}(E)=N$$

This equation expresses the idealised (minimum) costs or maximum cost benefit per patient in a defined group comprising healthy and unhealthy patients in monetary terms. Under such conditions, the costs that have been allocated for service provision can provide a completely accurate assessment of a group of patient's clinical status. Thus, the diagnostic cost–benefit outcome defined by Equation 4 may be expressed as a fraction of the idealised outcome given by Equation 5 and can be called the fractional net cost benefit per patient investigation.

Thus, when diagnostic uncertainty is present, represented by the false-positive and false-negative outcomes in Equation 4, the gross cost benefit is reduced compared with an idealised process. Not only are the average numbers of true diagnostic outcomes reduced for a given level of expenditure, but also there is an increased diagnostic uncertainty (cost detriment) associated with each diagnosis. Thus, with decreasing sensitivity and specificity, each diagnosis carries with it an increased uncertainty burden that erodes the beneficial cost utility (diagnostic strength) for individual patients. In effect, the cost of achieving a level of diagnostic accuracy is increased in proportion to the observed average numbers of unhelpful false diagnostic outcomes. These false-positive/false-negative outcomes will in practice arise from variations in

- the performance of the imaging system,
- the abnormalities present (size, extent, etc.),
- the observer performance in forming a diagnostic outcome.

The last two factors may in turn be affected by the degree of preselection by a medical practitioner of the patient population prior to referral, usually dictated by the extent of clinical symptoms or prior clinical knowledge. Consequently, the magnitude of the resultant diagnostic cost benefit arising from the true-positive/true-negative outcomes will be dependent upon the relative magnitudes of the terms within each square bracket in Equation 4. These are dictated by the sensitivity and specificity of the imaging process. Equation 4 may be expressed in terms of sensitivity and specificity by noting

$$\begin{array}{ll} & T/P+F/N=1\;\mathrm{and}\;T/N+F/P=1\\ & \mathrm{sensitivity}=\left(T/P\right)/(T/P+F/N)\;\mathrm{and}\\ & \mathrm{specificity}=\left(T/N\right)/\left(T/N+F/P\right) \end{array}$$

Hence, Equation 4 may be written as

(6) $$\begin{array}{ll} K(E) & =[N_{P}T/P(E)+N_{N}T/N(E)]\\ & \;-\{N_{P}T/P(E)\;[(1-\text{sens}/\text{sens}]\\ & \;+N_{N}T/N(E)[(1-\text{spec})/\text{spec}]\}-NR_{X}(E) \end{array}$$

As indicated previously setting sensitivity = specificity = 1 and *NR*_X_(*E*) ≪ *N*_P_  , *N*_N_ in Equation 6 leads to Equation 5, which gives the cost benefit of idealised (perfect) outcomes, where every patient receives a correct diagnosis.

The diagnostic uncertainty, which decreases the resultant cost benefit, may be due to any uncertainty or variation in the disease status within a patient population. Indeed, a number of studies have shown that the sensitivity and specificity of diagnostic tests appear to be dependent upon disease prevalence in the populations studied as well as its extent^(7–9)^. It is worth pointing out that any preselection process applied to a group of patients prior to referral for an X-ray examination is also a relevant cost overhead and should be included within the operating costs Op(*E*) in Equation 2. In fact, preselection that involves the use of imaging techniques, such as chest radiography prior to the use of computed tomography (CT) in screening for lung cancer, has been investigated^(10)^.

## WORKED EXAMPLES

In order to explore the effects of uncertainty in the diagnostic detection process, a patient population of 10,000 can be considered who undergo an examination on an imaging system with, for example, a sensitivity = specificity = 0.9 when the prevalence of disease varies. Assuming the sensitivity and specificity remain constant for the different levels of prevalence 1, 10, 30 and 50 %, it is possible to calculate the *T*/*P*(*E*) and *T*/*N*(*E*) values that underpin the gross benefit as well as the *F*/*P*(*E*) and *F*/*N*(*E*) values that underpin cost detriment and hence the overall net cost benefits (Table 1). This range of prevalence values spans the range from asymptomatic to highly symptomatic patient groups.

**Table 1.** Variation in detection outcomes for an imaging system with fixed sensitivity and specificity employed on a population of 10 000 patients with variable prevalence of disease.

Prevalence (%)	*T*/*P*(*E*)	*T*/*N*(*E*)	*F*/*P*(*E*)	*F*/*N*(*E*)	Fractional cost benefit
1	90	8910	990	10	0.8
10	900	8100	900	100	0.8
30	2700	6300	700	300	0.8
50	4500	4500	500	500	0.8

It is also possible to construct the detection outcome table for the patient population group with 30 % disease prevalence for investigations in which the sensitivity and specificity ratios vary from 0.5/0.95, 0.7/0.95, 0.8/0.8 and 0.9/0.7 (Table 2).

**Table 2.** Variation in detection outcomes for an imaging system with variable sensitivity and specificity employed on a population of 10 000 patients with 30 % disease prevalence.

Sensitivity/specificity	*T*/*P*(*E*)	*T*/*N*(*E*)	*F*/*P*(*E*)	*F*/*N*(*E*)	Fractional cost benefit
0.5/0.95	1500	6650	350	1500	0.63
0.7/0.95	2100	6650	350	900	0.75
0.8/0.8	2400	5600	1400	600	0.60
0.9/0.7	2700	4900	2100	300	0.71

Similarly, it is possible to construct the detection outcome table for the patient population group with 1 % disease prevalence (asymptomatic population) undergoing diagnostic investigations for which the sensitivity and specificity ratios vary from 0.5/0.95, 0.7/0.95, 0.8/0.8 and 0.9/0.7 (Table 3).

**Table 3.** Variation in detection outcomes for an imaging system with variable sensitivity and specificity employed on a population of 10 000 patients with a 1 % disease prevalence.

Sensitivity/specificity	*T*/*P*(*E*)	*T*/*N*(*E*)	*F*/*P*(*E*)	*F*/*N*(*E*)	Fractional cost benefit
0.5/0.95	50	9405	495	50	0.89
0.7/0.95	70	9405	495	30	0.89
0.8/0.8	80	7920	1980	20	0.60
0.9/0.7	90	6930	2970	10	0.40

Also shown in each table is the fractional net cost benefit arising from the diagnostic procedure for each patient population and sensitivity/specificity combination. In order to compare the numbers of false-positive and false-negative outcomes with the numbers of cancers induced, a detriment-adjusted nominal risk coefficient can be employed for cancer and inheritable effects of 5.7 × 10^−2^ Sv^−1^ proposed by ICRP^(11)^. If an average patient dose per examination of 1 mSv is assumed for the group of 10 000 patients, then *NR*_X_ (*E*) would be 0.57, and for 10 mSv average dose, it would be 5.7, hence in general *NR*_X_(E) ≪ *N*_P_, *N*_N_. The radiation cost detriment (expressed as the number of cancers induced) is only comparable with the false-negative [*F*/*N*(*E*)] rate for patient groups with a low prevalence of disease who undergo a relatively high-average-dose examination with high sensitivity. However, it would still be much lower than the false-positive [*F*/*P*(*E*)] rate even for high specificity (Table 3).

## DISCUSSION

The results presented in Table 1 demonstrate that irrespective of disease prevalence the fractional net cost benefit is always the same when sensitivity = specificity. Thus, in Table 1, the fractional net cost benefit is 80 % of an ideal diagnostic process even when sensitivity = specificity = 0.9. Under these circumstances, only 90 % of both the prevalent disease and non-disease conditions are accurately diagnosed with 10 % of each misdiagnosed. Thus, the overall effective costs of the true-positive/true-negative outcomes are increased by 1/0.8 = 1.25 due to the non-productive diagnoses. However, when sensitivity and specificity differ, the fractional net cost benefit will depend upon the prevalence (cf. Tables 2 and 3).

Within an overall framework of healthcare economics the false-positive and false-negative diagnoses also contribute an extra cost burden, which is transferred outside the primary diagnostic cost regime. The false-positive diagnoses will either necessitate further investigation (imaging/biopsy) in order to obtain verification or even lead to unnecessary clinical interventions. The actual numbers of these false-positive outcomes, for a given level of sensitivity, will vary with the disease prevalence. Equally the numbers of false-negative outcomes also vary with disease prevalence. The latter outcome may well lead to (a) a patient presenting for an examination at a future date with a higher degree of morbidity or (b) death.

Both of these outcomes (*F*/*P*(*E*), *F*/*N*(*E*)) represent a significant cost detriment that may far exceed that arising from the radiation risk associated with the use of ionising radiation. This is the case even when a high degree of referral preselection of the patient population has occurred (50 % prevalence group). Consequently, any cost savings that might result at the referral stage by immediately and automatically referring a patient for an X-ray examination may lead to significant cost increases in the future when relatively large numbers of false-positive/false-negative outcomes may result. Incidentally, it has been pointed out by ICRP that during the period that the Publication 103 recommendations in respect of cancer risk are likely to apply, the survival rates for many cancers are expected to rise^(11)^.

For a given prevalence, results presented in Tables 2 and 3 demonstrate that the fractional net cost benefit varies with the sensitivity and specificity. In particular, for 30 % prevalence, where 70 % of the patient population is disease free, it is the specificity that has most effect. Under these circumstances, when the specificity drops to 0.7, a very large number of false-positive outcomes are predicted (2100 per 10 000) with their associated cost detriment (Table 2). However, the number of false-positive outcomes increases markedly (2970 per 10 000) when the prevalence drops to 1 % (Table 3), for the same specificity and the fractional net cost benefit is decreased still further or the cost of true-positive/true-negative outcomes is increased significantly (i.e. by 1/0.43 = 2.3X).

Accurate diagnosis plays a vital role in the overall cost–benefit/cost–detriment framework even with a high degree of referral preselection of a group of patients when it is assumed that an examination is fully justified. If the patient group represented by 1 % prevalence shown in Table 3 is part of a screening programme, then obviously it is desirable to have both sensitivity and specificity as high as possible. Then both true-positive and true-negative outcomes would be high and the associated cost detriment arising from false-positive/false-negative outcomes low. However, it can be deduced that a sensitive examination is more valuable when false-negative outcomes are more undesirable than false-positive ones. However, high specificity is more valuable if false-positive outcomes are more undesirable.

X-ray examinations applied to a low-prevalence group of patients that is not part of a screening programme will spend much of its resources in verifying negative disease status (the worried well syndrome) in order to detect a relatively low number of positive outcomes (Table 3). Equally, when the sensitivity or specificity = 0.5 the true-positive or true-negative outcomes are matched or neutralised by equal numbers of false-negative or false-positive outcomes, respectively, and the net diagnostic cost benefit is zero. The diagnostic process is then operating under conditions of maximum uncertainty and essentially providing random outcomes operating on the diagonal of the appropriate receiver operating characteristic (ROC) space.

### Sensitivity and specificity in diagnostic radiology

What are typical sensitivities and specificities arising from diagnostic X-ray examinations? A detailed study of the role of digital chest radiography in the screening for lung cancer demonstrated the sensitivities and specificities that might be expected from radiographic examinations as well as the role of observer performance in determining these outcome measures^(10)^. This study concluded that a detection rate of 94 % for lung tumours with a diameter in the range of 6.8–50.7 mm (as verified by CT) was achievable with chest radiography only at the expense of a high false-positive rate requiring an excessive number of workup CT examinations.

Typically, over 50 workup CT examinations (false positives) were required per cancer detected in order to achieve a sensitivity of ∼70 % and specificity <50 % for digital radiography. However, detection performance was strongly observer dependent. Instances in which chest radiography alone could be relied upon showed some 22–63 % of lung cancers would be missed at a stage of disease at which they could be detected with CT. Cancer prevalence in the study group was 1.3 %. Consequently, a higher-dose/higher-cost CT examination provides significantly higher sensitivity and specificity than a lower-dose/lower-cost radiographic examination.

This comparison of the diagnostic performance of radiographic and CT techniques clearly demonstrates the significantly higher sensitivity and specificity of CT methods. The results help to explain (albeit retrospectively) why radiologists have voted with their diagnostic feet in moving over to CT examinations during the past 25 y. Such an improvement could only be achieved through increased costs and patient doses. However, whether this has been achieved cost-beneficially for all patient populations and examinations is still unclear. Equally, it is also unclear whether present attempts to lower patient doses in CT examinations will maintain sensitivity and specificity and, therefore, constant net cost benefit.

A detailed review of the justification of CT for individual health assessment has been undertaken in the UK by a working party of the Department of Health^(12)^. The report reviewed applications in lung cancer, colorectal cancer and polyp detection as well as coronary heart disease.

In the case of lung cancer screening, the prevalence detected in all non-randomised trials was 0.5–2.7 %, with 4–33 % (false positives) of these undergoing interventions for benign disease. The corresponding sensitivity and specificity were not discussed; however, it was indicated that for lung cancer detection rate of roughly 1 % the probability of detecting benign lesions (false positive) was 50× higher. It was indicated that if multidetector CT (MDCT) was employed that very few lung cancers are missed but at a price of over diagnosis. Specific follow-up scanning regimes are employed in order to minimise unnecessary interventional procedures. Important final considerations included the identification of unimportant disease (pseudo true positive), the failure to identify important disease successfully and the expenditure of money that may be better utilised elsewhere.

Colorectal cancer and polyp ≥10 mm were detected significantly more frequently by CT compared with barium enema (7.4 versus 5.6 %). Also, in follow-up studies, it was found that barium enema had twice the miss rate (14 versus 7 %). A statistical analysis of multi-trial results (49 studies) indicated that CT colonography had a sensitivity of 94 % for cancer detection, comparable with colonoscopy. In a US study, a mean per patient sensitivity of 90 % and specificity of 86 % for cancer and large polyps (>10 mm) was also noted. These figures were similar to those observed in other studies. The requirement for adequate training for reporting radiologists was an important outcome.

The specificity of CT calcium scoring and CT coronary angiography were indicated to be extremely high (95–100 and 97–100 %, respectively). Thus, the numbers of false-negative outcomes even for asymptomatic self-referred individuals would remain low. Thus, justification mechanisms may need to be disease dependent and in certain instances self-referral could be quite acceptable.

A comparison of digital breast tomosynthesis (DBT) with full-field digital mammography (FFDM) has been undertaken as part of the UK Breast Screening Programme (TOMMY) trial^(13)^. Results indicated that 2D FFDM had a sensitivity of 87 % and a specificity of 59 %, 2D FFDM with DBT had a sensitivity of 89 % and a specificity of 69 %. Finally synthetic 2D, reconstructed from the DBT images, together with DBT provided a sensitivity of 88 % and a specificity of 71 %.

A recent comparison of mammography screening programmes in the USA versus Denmark has highlighted the much higher recall rate (lower specificity) in the US programme^(14)^. Only 1 out of 21 women recalled for workup had breast cancer in the USA (specificity 83.2 %) with an equivalent figure of 1 out of 3 in Denmark (specificity 96.6 %). It was concluded that the higher cost detriments associated with the higher recall rates, as well as the increased anxiety for the women involved, merit further consideration.

### Application to radiation protection

The ethical basis for justification is presently a major consideration within the medical radiation protection field with the reduction or even elimination of unnecessary exposure a major driving force. Whilst important, this approach does not take full cognisance of the role and relevance of diagnostic risk and its fundamental role in justification. It is clinical outcomes from diagnostic X-ray examinations that establish the primary basis for justification. Both diagnostic cost benefit and cost detriment can vary significantly, depending upon the sensitivity and specificity of a diagnostic procedure applied to a given group of patients. Lower patient dose may produce a lower cost detriment due to the radiation risk; however, it may be associated with an increase in diagnostic cost detriment due to a lower sensitivity and specificity. In fact, true optimisation cannot be assessed or verified without knowledge of diagnostic performance.

In the UK, ∼41.5 million medical and dental X-ray examinations are performed each year resulting in a per caput effective dose of 0.33 mSv**^(^**^15^**^)^**, which corresponds to a population dose of 13 695 manSv. If a lifetime risk of cancer induction of 5.7 × 10^−2^ per Sv^(11)^ is assumed, then this annual effective dose indicates that on average 781 cancers would be induced, many of which would not present during a patient's lifetime. Also, if it is assumed that all X-ray examinations are undertaken with high sensitivity = specificity = 0.95 on a population with an average prevalence of disease of 30 %, then the number of false-positive outcomes would be 1.45 × 10^6^ and the number of false-negative outcomes 0.62 × 10^6^. All of these outcomes could have some degree of immediate clinical impact.

The frequency of X-ray examinations and associated per capita effective dose are relatively low in the UK. For Europe as a whole (population roughly 500 million) on average, every person receives an X-ray examination each year with an average per capita dose of 1 mSv^(16)^. For a 30 % prevalence and 95 % detection accuracy, the annual number of false-positive outcomes would be 17.5 million and false-negative outcomes 7.5 million. The corresponding average number of cancers induced per annum would be 28 386.

For all economic Level 1 countries in the world, the total number of medical and dental X-ray examinations each year is roughly 2.4 × 10^9^, which employ a total collective effective dose of 2.9 × 10^6^ manSv^(17)^. If the diagnostic accuracy was 95 % (sensitivity = specificity = 0.95), then ∼1.2 × 10^8^ incorrect diagnoses and 1.65 × 10^5^ induced cancers would be indicated. The diagnostic accuracy (sensitivity and specificity) that would be required to ensure that the number of false diagnostic outcomes (diagnostic cost detriment) equalled the number of cancers induced (radiation cost detriment) in this population would need to be 99.9934 %.

It is perhaps worth noting that for all economic Level 1 countries, the possible 1.20 × 10^8^ incorrect diagnoses (roughly equivalent to the combined population of the UK and France) with a per capita dose of 1.9 mSv^(17)^ indicates that ∼13 537 cancers could be induced within this sub-group of patients. However, examinations with incorrect outcomes could not be deemed to fulfil the basic principles of radiation protection.

ICRP has established three levels of justification of a radiological practice in medicine:

- At the first and most general level, the proper use of radiation in medicine is accepted as doing more good than harm to society.
- At the second level, a specified procedure undertaken on a group of patients with a specified objective is defined and justified.
- At the third level, the application of the procedure to an individual patient should be judged to do more good than harm (to that patient).

An examination that does not provide more good than harm to an individual patient (Level 3 justification) could not be classed as justified unless justification itself had been linked to accepted and defined statistical variations in diagnostic performance for a group of patients for a particular type of examination (Level 2 justification). In fact, the NCRP efficacy model with its inherent statistical variations in clinical outcomes underpins the whole framework of justification, and therefore, defined and accepted variations in diagnostic performance should be fundamental aspects of radiation protection of the patient. Equally, false diagnostic outcomes could not be considered part of an optimised process unless acceptable, ‘optimised’ statistical variations in diagnostic outcomes have been defined and accepted.

It is impractical to expect 100 % correct diagnosis from any examination; however, controlled and quantifiable uncertainty would seem to be a desirable goal. Equally, although these undesirable outcomes are a relatively small fraction of the overall patient population who undergo X-ray examinations, the associated cost detriment, expressed as the number of undesirable diagnostic outcomes, would appear to be significantly greater than that resulting from the radiation risk. Thus, from an economic and associated diagnostic risk perspective, the optimisation of diagnostic cost benefit and minimisation of associated cost detriments are a priority.

The proposed fractional net cost benefit per patient examined arising from diagnostic investigations on a representative patient population can be used as a quantitative measure of both justification and optimisation. It expresses the observed cost benefit relative to an ideal diagnostic process and incorporates all the elements of Level 1 technical/equipment performance efficacy with those of Level 2 diagnostic accuracy efficacy proposed in the NCRP efficacy model. The approach adopted takes full cognisance of false-positive and false-negative outcomes and assesses diagnostic performance in terms of quantities that can be related directly to economic performance via the cost–benefit model suggested by ICRP as a basis for underpinning ALARA and justification^(3)^.

## SUMMARY AND CONCLUSIONS

A radiation risk-driven framework for radiation protection of the patient in X-ray diagnosis, whereby the lowering of doses is the primary consideration, is inadequate, when technological developments not only affect patient doses but also information gained and hence diagnostic performance. Justification of medical exposures is based primarily on diagnostic performance, which can be defined by the sensitivity and specificity of a diagnostic examination.

Application of the cost–benefit model proposed by ICRP demonstrates that the magnitude of the diagnostic cost detriment represented by the numbers of false-positive and false-negative outcomes may be significantly greater than the numbers of induced cancers predicted, even when optimistic values of sensitivity and specificity are assumed. Such incorrect outcomes may have immediate clinical impact, for example with patients undergoing diagnosis for cancer. However, radiation-induced cancers may not present for an extended period or even not at all in a patient's lifetime. That is not to say that the judicious and optimum use of ionising radiation is irrelevant, it should be a fundamental requirement of high-quality, patient-centred diagnostic services. Nonetheless, a large number of incorrect diagnoses may occur worldwide with no apparent benefit to the patients concerned. Such examinations cannot fulfil the basic principles of radiation protection yet are subject to an associated radiation detriment.

The increase in per capita doses arising from the diagnostic use of ionising radiation in medicine in developed nations over the past three decades has been driven by an obvious desire by the medical community for improved diagnostic accuracy, provided by ongoing developments in CT technology. Unfortunately, it is not yet known whether such increases in patient doses have always been in proportion to any improvements in diagnostic outcomes.

Analyses of diagnostic outcomes arising from screening programmes are now ongoing throughout Europe and North America. However, analysis of diagnostic outcomes arising from routine practice is now feasible given the widespread application of picture archiving and communication systems (PACS) and associated information technology (IT) systems to healthcare and the corresponding potential for time-dependent population studies. Widespread analysis and intercomparison of the numbers of positive and negative diagnostic outcomes resulting from particular examinations and patient populations would help establish both the cost effectiveness of radiological service provision and its diagnostic efficacy.

## FUNDING

Funding to pay the Open Access publication charges for this article was provided by Integrated Radiological Services (IRS) Ltd.
